# The effect of reimbursement on MRgFUS treatment of uterine fibroids

**DOI:** 10.1186/2050-5736-3-S1-O93

**Published:** 2015-06-30

**Authors:** Ronit Machtinger, Yael Inbar, Aviva Alagem-Mizrachi, Jaron Rabinovici

**Affiliations:** 1 Sheba Medical Center, Tel Hashomer, Israel

## Background/introduction

The demand for conservative treatments for uterine fibroids has increased during the last decade, as more women wish to retain their uterus and avoid invasive procedures. MRgFUS is a well established conservative technology for the treatment of uterine fibroids. The procedure is offered as a clinical treatment option (not as a part of clinical trials) at the Sheba Medical Center for many years, and was mostly self-paid by the patients. From November 2013, the procedure has been covered by the HMOs as a part of the Israeli National Health Insurance Law. The aim of the study was to assess how reimbursement affects the number of MRgFUS enrollments and treatments in a single tertiary center.

## Methods

A retrospective analysis of the number of patients attending MRgFUS clinics before (December 2012 to February 2013, period I) and after reimbursement (December 2013 to February 2014, period II). For statistical analysis Fisher exact test was performed.

## Results and conclusions

In period I, 15 women out of 20 (75%) fulfilled basic MRgFUS criteria and were referred for MRI. Twelve MRI, 7 were found suitable and 3 were ultimately treated. In period II, 39 women out of 63 (61.9%) fulfilled basic MRgFUS criteria and were referred for MRI. Thirty performed MRI, 12 were found suitable all underwent MRgFUS treatment. The number of treated patients was significantly higher following the reimbursement (p<0.001).

Conclusions: Reimbursement significantly increased the number of patients who attended the MRgFUS clinics and quadrupled the number of treatments.

